# DETECTING STRESS INCIDENCE WITH WRISTBAND SENSOR-BASED DEVICE: A WAY TO SUPPORT CAREGIVERS OF PERSONS WITH DEMENTIA

**DOI:** 10.1093/geroni/igz038.1433

**Published:** 2019-11-08

**Authors:** Angela YM Leung

**Affiliations:** Centre for Gerontological Nursing, School of Nursing, The Hong Kong Polytechnic University, Hong Kong, Hong Kong

## Abstract

This experimental study aimed to investigate the sensitivity of detecting stress level with a wearable device. A total of 10 caregivers underwent a psychological stress test with a wearable sensor-based device that measured skin conductivity (Electrodermal Activity, EDA) and heart rate variability (Interbeat Interval, IBI). Two sample t-tests were used to compare the mean scores of EDA and IBI at baseline and during stress. The accuracy for classifying stress test epochs was 70%. There was a significant increase in EDA at the time when the participants were under stress [mean score (SD) at baseline vs at stress: 0.089 (SD 0.150) vs 0.427 (SD 0.430), p <0.001]. However, insignificant changes in IBI were observed. In conclusion, this wearable sensor-based device was able to recognize stress incidence and accurately capture physiological changes. It has a potential to be part of the sensor-based stress monitoring and alleviating system (SbSMA).

